# Prevalence and Associated Factors of Osteoporosis Among People With Hypertension in Alahsa, Saudi Arabia: A Cross-Sectional Study

**DOI:** 10.7759/cureus.66961

**Published:** 2024-08-15

**Authors:** Hussain Aldakhlan, Rahma Baqer, Mohammed Alramdan, Abdullah Albinsaleh, Fatimah Albesher, Zakaria Alsharidah, Habeeb Alabdullah

**Affiliations:** 1 Al-Ahsa Health Cluster, Saudi Board of Preventive Medicine, Al-Ahsa, SAU; 2 Community Wellness Department, Al-Ahsa Health Cluster, Ministry of Health, Al-Ahsa, SAU; 3 Department for Communicable Disease Control, Al-Ahsa Health Cluster, Ministry of Health, Al-Ahsa, SAU

**Keywords:** saudi arabia, alahsa, associated factors, osteoporosis, hypertension, prevalence

## Abstract

Introduction

Osteoporosis (OP) and hypertension (HTN) are prevalent conditions impacting elderly health. This study aimed to explore the prevalence and factors associated with OP among people with HTN in Al-Ahsa, Saudi Arabia (2023).

Material and method

A cross-sectional analytical study was conducted. Adults aged 50-79 diagnosed with HTN were recruited from those referred for dual-energy X-ray absorptiometry (DEXA) scans using a systematic random sampling method. The participants' electronic health records were reviewed and all participants were interviewed using a structured questionnaire to collect data not available in the electronic health records related to demographics, medical history, and lifestyle factors. Chi-square tests and multivariable logistic regression were used to assess the associations between OP and clinical parameters.

Results

A total of 255 participants were recruited, with 115 (45.1%) having normal bone density, 97 (38%) having osteopenia, and 43 (16.9%) having OP. Females 167 (65.5%) were higher than males 88 (34.5%). The average age of all the respondents was 66.2 ± 7.96 years, and their average body mass index (BMI) was 22.2 ± 15.1. The age in years (mean ± SD) of participants with OP 68.04 ± 7.60 was higher compared to normal 64.9 ± 7.46 (p-value = 0.03). Factors that appear to increase the risk of OP in multivariable logistic regression analysis with an adjusted odds ratio (OR) (95% CI) include increased age (OR: 1.17, CI: 0.9-1.2, p-value = 0.048), and parathyroid gland disorder comorbidity (OR: 15.1, CI: 0.7-32, p-value = 0.03), while some factors that reduce the risk of developing OP include increased BMI (OR: 0.9, CI: 0.91-1.03, p-value = 0.03), literate individuals (OR: 0.1, CI: 0.01-1.4, p-value = 0.046), and taking beta-blockers (BB) treatment (OR: 0.23, CI: 0.01-1.3, p-value = 0.02) reduced odds of developing OP according to results.

Conclusion

The OP is notably present among people with HTN, especially older people, and parathyroid gland disorders. Higher BMI levels, along with the use of BB, help to decrease it. Additionally, any level of education above illiteracy is associated with a lower prevalence of OP, suggesting that education may have a protective effect against OP in HTN patients. We recommend further research on OP risk factors in HTN Saudi patients. Future research should focus on assessing the impact of educational levels and socioeconomic factors on OP prevalence and investigating the association between specific comorbidities (e.g., diabetes mellitus (DM) and parathyroid gland disorders) and OP risk in HTN individuals. Collaborate with public health authorities and organizations to integrate OP screening into routine HTN patient care protocols.

## Introduction

According to the World Health Organization (WHO), osteoporosis (OP) is a disease characterized by low bone mass and deterioration of bone tissue architecture, leading to increased bone fragility and fracture risk [1]. HTN, on the other hand, is defined by the WHO as the current use of blood pressure-lowering medication associated with an average systolic blood pressure ≥140 mm Hg and an average diastolic blood pressure ≥90 mm Hg or an average systolic blood pressure ≥130 mm Hg and an average diastolic blood pressure ≥80 mm Hg (if they had a history of cardiovascular disease or DM) [2].

In Saudi Arabia’s general population, the prevalence of HTN was 17.8% among men and 12.5% among women [3]. According to 2018 epidemiological data for Saudi Arabia, the prevalence of OP was 28.2% among men and 37.8% among women between the ages of 50 and 79 years [3,4]. Several studies from different parts of the world explored the pathophysiology that correlates HTN with OP, such as age, gender, smoking status, low calcium levels and vitamin D deficiency [3]. To the best of our knowledge, Saudi Arabia has not published enough data about the correlation between HTN and OP [3].

Several studies have indicated a probable close relationship between cardiovascular disease and OP, particularly in older individuals. Low bone density is an independent risk factor for cardiovascular disease mortality, with women having a higher risk [1]. Additionally, it has been noted that there is a significant correlation between risk factors for cardiovascular disease and the existence of vertebral fractures [1]. According to earlier research, HTN significantly raises the risk of OP and osteoporotic fracture and refracture, ultimately raising the rate of mortality [5].

OP and HTN are prevalent conditions that negatively impact the health-related quality of life among the elderly. On the other hand, research on the connection between OP and HTN is scarce. The American National Foundation recommends DEXA of the hip, forearm, and spine with a T-score of ≤ −2.5 to quantify bone mineral density (BMD) to diagnose OP [3].

The literature explores the pathophysiology of the relationship between OP and HTN and the factors linking OP and HTN: chronic elevation in the levels of parathyroid hormone (PTH), angiotensin II, and catecholamines, including adrenaline in HTN, which may have an impact on bone health. Furthermore, renal calcium excretion will decrease vitamin D plasma concentrations. Simultaneously, HTN is associated with reduced intestinal absorption of calcium and vitamin D, all of which contribute to the continuous secretion of PTH. Moreover, persistent PTH elevation increases osteoclast activity, which aids in bone resorption [2].

Antihypertensive medication, particularly BB, affects bones to ascertain whether or not it can increase BMD. A study of postmenopausal Syrian women did not find a correlation between HTN and OP. This study demonstrates that BB and thiazides have beneficial effects on the BMD of the lumbar spine in osteoporotic HTN postmenopausal women [6].

Another study examined the impact of loop diuretics and thiazides on vertebral fracture incidence following stroke. While short-term use was associated with a higher incidence of vertebral fracture, further research is needed to confirm this finding, considering vertebral fracture as a common site of osteoporotic fracture [7].

Sympathetic tone, a consistent factor in HTN, may contribute to bone loss through increased sympathetic outflow and beta-adrenergic stimulation. Antihypertensive medications that block alpha and beta-adrenergic receptors could potentially mitigate bone loss while reducing blood pressure [8].

HTN-induced immune system activation produces cytokines such as interleukin 17A (IL.17A), tumor necrosis factor-alpha (TNF-alpha), and interleukin 6 (IL6), which accumulate in the bone marrow and kidney. Interleukin 17A stimulates osteoclast activity via the receptor activator of nuclear kappa B ligand (RANKL), leading to bone resorption [8].

A few cross-sectional studies reported an inverse relationship between HTN and the level of vitamin D (10 ng). An increased level of 25-hydroxy vitamin D (25OH) was associated with a 12% decrease in blood pressure readings [8].

Ye et al. conducted a systematic review and meta-analysis in China [9] to evaluate the association between HTN and BMD. The results demonstrate that HTN can reduce the BMD in the following areas: the lumbar spine, femoral neck, Ward's triangle, femoral intertrochanteric, calcaneus, and distal forearm [9]. This meta-analysis suggests that HTN can reduce the BMD of the human body, and for different parts of the bone, the degree of reduction is different according to the bone site [9].

To the best of our knowledge, Saudi Arabia has not published enough data about the correlation between HTN and OP. Still, there is a deficiency in the data that explores the shared risk factors linked to HTN and OP. Our aim of this study is to explore the prevalence and factors associated with OP among HTN patients in Al-Ahsa, Saudi Arabia, in 2023. Understanding the prevalence of OP among HTN patients in Al-Ahsa, Saudi Arabia, is crucial for developing targeted prevention and management strategies. This region's specific population demographics and potential risk factors necessitate investigating this association.

## Materials and methods

Study design

This research adopts an analytical cross-sectional approach, investigating patients with documented HTN from the Saudi Ministry of Health electronic databases.

Study population

This study population included records of adults aged 50 to 79 diagnosed with HTN who attended primary healthcare centers and registered in the Alahsa community health administration databases between January 2021 and October 2023. Criteria include age, gender, Saudi citizenship, confirmed diagnoses of HTN, and having undergone a DEXA scan. Pregnant females and patients with missing data were excluded from this study.

Sample size

According to a published study, the prevalence rate of OP in Alahsa city is 34% [10]. Using Epi Info software, we calculated a representative sample size. Assuming a confidence level of 95% and a margin of error of 5%, we determined the required sample size to be 345 participants [11].

Sampling technique

The study population comprised 1,745 male and female patients over 50 years old, referred from Al-Ahsa's four primary healthcare sectors to the Al Jafar Center for OP screening via DEXA scan. We selected 345 participants from the records of patients referred to the Al Jafar Center using systematic random sampling, ensuring that each patient had an equal chance of inclusion in the study. Most of the data were primarily collected from the Saudi Health Electronic Medical Records (EMRs); this included I.D., demographic data, medications, and confirmed diagnoses of HTN retrieved from EMRs using ICD-10 codes for HTN. BMD measurements from DEXA scans are used to identify normal osteopenia, or OP, and provide relevant laboratory results for all participants.

For all participants, some missing data were not available in the EMR, so we constructed a standardized questionnaire (Appendix 1). This questionnaire was developed based on a comprehensive literature review on OP and HTN and associated factors, and the content validity of the questionnaire was revised by two consultants, one in preventive medicine and one in family medicine. We conducted pilot studies with 10 volunteer participants who were not included in the study sample to assess the clarity and feasibility of this questionnaire. The questionnaire was administered via telephone to collect the missing information from the electronic records of all participants. Trained medical professionals followed standardized administration procedures to minimize bias and ensure data completeness.

Study variables

The variables selected to determine the prevalence of OP and its associated factors with HTN include demographic data (age, gender, education level, occupation, and marital status), lifestyle factors (such as cigarette smoking), and medical history variables (including HTN duration, obesity, age at menarche, duration of menopause, comorbidities, and the use of antihypertensive medication).

Measurement of the outcome

OP was defined according to the WHO criteria, with diagnosis confirmed by a T-score of -2.5 or lower in the lumbar spine, femoral neck, or hip as assessed by DEXA scans. BMD status, categorized as normal, osteopenia, or OP, served as the primary outcome variable. A T-score greater than −1 was considered normal; a T-score between −2.5 and −1 indicated osteopenia; and a T-score of −2.5 or lower indicated OP. We assigned each participant to a BMD status category based on their hip BMD T-scores.

Response rate

Our response rate was 73.9%, with 255 out of 345 participants responding. During data collection, several challenges impacted our response rate: 28 participants had passed away, 8 lacked ID numbers for data linkage, 15 had missing contact details, 12 refused to participate, and 27 did not respond despite three attempts to contact them.

Statistical analysis

Two software programs, SPSS for Windows version 21 (SPSS, Chicago, IL, USA) and GraphPad Prism version 8.4.2 (GraphPad Software, San Diego, CA, USA), were used to perform the statistical analysis. The Kolmogorov-Smirnov test was performed to check the normality of the variables. Descriptive statistics were expressed as mean and standard deviation, or frequency and percentage, according to the type of data. A chi-square test and one-way ANOVA test were conducted to assess the association between BMD and clinical parameters. Multivariable logistic regression analysis was applied to further examine the correlation between parameters and osteoporosis. A P-value < 0.05 was considered statistically significant.

## Results

This study recruited 255 participants with HTN who underwent BMD. The ratio of females 167 (65.5%) was higher than that of males 88 (34.5%). The average age of all the respondents was 66.2 ± 7.96 years, and their average BMI was 22.2 ± 15.1. Among females, 76 (29.8%) had menarche at the age of 12 to 13 years. Menopause age occurred between 41 and 50 years; 60 (23.5%) and 61 (23.9%) faced menopause at 51 to 55 years, and only 2 (0.8%) females did not face menopause yet. The majority of the respondents were married, 200 (78.45%), and non-smokers, 235 (92.2%). Additionally, 102 (40%) of the respondents were illiterate, and 151 (59.2%) of the females were housewives. Approximately half of the respondents were diabetic patients; 118 (46.3%) and 119 (46.7%) had no comorbidities (Table 1). Out of the participants, 115 (45.1%) had normal BMD results, 97 (38%) had osteopenia, and 43 (16.9%) had OP (Table 2).

**Table 1:** Demographic characteristics of HTN patients (n=255) Data are presented as numbers, percentages, mean, and standard deviation (mean ± SD). HTN: hypertension; BMI: body mass index.

Variables	N (%)
Gender	
Female	167 (65.5%)
Male	88 (34.5%)
Age in years (mean ± SD)	66.2 ± 7.96
BMI (mean ± SD)	22.2 ± 15.1
Menarche age	
Less than 10 years	12 (4.7%)
10–11 years	33 (12.9%)
12–13 years	76 (29.8%)
14 years	11 (4.3%)
More than 14 years	8 (3.1%)
N/A	115 (45.1%)
Menopause age	
35–40 years	10 (3.9%)
41–50 years	60 (23.5%)
51–55 years	61 (23.9%)
55–60 years	15 (5.9%)
N/A	107 (42%)
Not yet	2 (0.8%)
Marital status	
Single	3 (1.2%)
Married	200 (78.4%)
Divorced	6 (2.4%)
Widow	46 (18%)
Smoking status	
Smokers	20 (7.8%)
Non-smokers	235 (92.2 %)
Level of education	
Primary	47 (18.4%)
Middle	19 (7.5%)
High	21 (8.2%)
University	11 (4.3%)
Illiterate	102 (40%)
Literate	55 (21.6%)
Occupation	
Government employee	8 (3.1%)
Private employee	7 (2.7%)
Retired	78 (30.6%)
Housewife	151 (59.2%)
Business owner	11 (4.3%)
Comorbidities	
Cancer	4 (1.6%)
Diabetes	118 (46.3%)
Renal diseases	1 (0.4%)
Parathyroid gland disorders	3 (1.2%)
Diabetes and parathyroid gland disorders	7 (2.7%)
Diabetes and renal diseases	3 (1.2%)
Nothing	119 (46.7%)

**Table 2:** Prevalence of BMD in HTN patients Data are presented as numbers and percentages. BMD: bone mineral density; HTN: hypertension.

Variable	N (%)
BMD	
Osteoporosis	43 (16.9%)
Osteopenia	97 (38%)
Normal	115 (45.1%)

All the participants who were included in this study were HTN patients. Among them, 184 (72.2%) were diagnosed with HTN between 40 and 60 years old. Out of 255 participants, 43 (16.9%) had calcium channel blockers (CCB) as their antihypertensive drug. Following CCB, 38 participants (14.9%) were taking angiotensin-converting enzyme inhibitors (ACE-I) (Table 3).

**Table 3:** Distribution of age at HTN diagnosis and antihypertensive drug use Data are presented as numbers and percentages. HTN: hypertension; BMD: bone mineral density; ACE-I: angiotensin-converting enzyme inhibitors, ARBs: angiotensin receptor blockers; BB: beta-blockers; CCB: calcium channel blockers.

Variables	N (%)
Age of HTN diagnosis	
40–60 years	184 (72.2%)
Less than 40 years	42 (16.5%)
More than 60 years	29 (11.4%)
Antihypertensive drugs (only one)	
ACE-I	38 (14.9%)
ARBs	32 (12.5%)
BB	20 (7.8%)
CCB	43 (16.9%)
Thiazide	8 (3.1%)
Antihypertensive drugs (combination)	89 (34.9%)

The association between BMD and demographic characteristics was analyzed. The age of HTN patients significantly differed among those with normal BMD, osteopenia, and OP. The mean age of patients with OP was significantly higher compared to those with normal BMD (age in years mean ± SD, 68.04 ± 7.60 years vs. 64.9 ± 7.46 years, respectively; p-value = 0.03). Additionally, the level of education showed a statistically significant association with OP: 26 out of 102 (25.5%) illiterate HTN patients experienced OP; conversely, 32 out of 55 (58.1%) literate HTN with normal BMD and only 7 out of 55 (12.7%) had OP, and 7 out of 11 corresponding to 63.6% HTN patients with normal BMD had attended university, and only 1 out of 11 (9%) HTN patients with OP had attended college (p = 0.018). Any level of education above illiteracy is associated with a lower prevalence of OP, suggesting that education may have a protective effect against OP in HTN patients. All other demographic parameters did not show any significant association (Table 4).

**Table 4:** Demographic characteristics of HTN patients with BMD Data are presented as numbers, percentages, mean, and standard deviations (mean ± SD). The chi-square test and one-way ANOVA test were used. *p<0.05 was considered statistically significant. HTN: hypertension; BMD: bone mineral density; BMI: body mass index.

Variables	Normal	Osteopenia	Osteoporosis	P-value
Gender				
Male	46 (52.3%)	32 (36.3%)	10 (11.4%)	0.078
Female	69 (41.3%)	64 (38.3%)	34 (20.4%)	
Age in years (mean ± SD)	64.9 ± 7.46	67.3 ± 8.85	68.04 ± 7.60	0.03*
BMI (mean ± SD)	23.9 ± 14.9	21.8 ± 14.8	18.4 ± 15.1	0.1
Menarche age				
Less than 10 years	3 (25%)	6 (50%)	3 (25%)	0.19
10–11 years	12 (36.4%)	13 (39.4%)	8 (24.2%)	
12–13 years	38 (50%)	28 (36.8%)	10 (13.2%)	
14 years	7 (63.6%)	3 (27.3%)	1 (9.1%)	
More than 14 years	2 (25%)	5 (62.5%)	1 (12.5%)	
Menopause age				
35–40 years	4 (40%)	3 (30%)	3 (30%)	0.25
41–50 years	26 (43.3%)	22 (36.7%)	12 (20%)	
51–55 years	24 (39.3%)	27 (44.3%)	10 (16.4%)	
55–60 years	8 (53.4%)	5 (33.3%)	2 (13.3%)	
Smoking status				
Smokers	6 (30%)	10 (50%)	4 (20%)	0.36
Non-smokers	109 (46.4%)	87 (37%)	39 (16.6%)	
Level of education				
Primary	27 (57.4%)	13 (27.7%)	7 (14.9%)	0.018*
Middle	13 (68.4%)	5 (26.3%)	1 (5.3%)	
High	9 (42.8%)	11 (52.4%)	1 (4.8%)	
University	7 (63.6%)	3 (27.3%)	1 (9.1%)	
Illiterate	34 (33.3%)	42 (41.2%)	26 (25.5%)	
Literate	32 (58.1%)	16 (29%)	7 (12.7%)	
Occupation				
Government employee	2 (25%)	4 (50%)	2 (25%)	0.5
Private employee	3 (42.9%)	3 (42.9%)	1 (14.2%)	
Retired	18 (23.1%)	42 (53.8%)	18 (23.1%)	
Housewife	9 (6%)	90 (59.6%)	52 (34.4%)	
Business owner	1 (9.1%)	8 (72.7%)	2 (18.2%)	
Comorbidities				
Cancer	2 (50%)	1 (25%)	1 (25%)	0.113
Diabetes	62 (52.5%)	41 (34.7%)	15 (12.8%)	
Parathyroid gland disorders	0	0	3 (100%)	
Diabetes and parathyroid gland disorders	3(42.9%)	3 (42.9%)	1 (14.2%)	
Diabetes and renal diseases	2(66.7%)	0	1(33.3%)	
Nothing	45 (37.8%)	50 (42%)	24 (20.2%)	

HTN patients showed insignificant results when BMD categories were analyzed against age of HTN diagnosis, one antihypertensive drug, and drug combination (Table 5).

**Table 5:** Clinical characteristics of HTN patients with BMD Data are presented as numbers, percentages, mean and standard deviations (mean ± SD). The chi-square test and one-way ANOVA test were used. HTN: hypertension; BMD: bone mineral density; ACE-I: angiotensin-converting enzyme inhibitors; ARBs: angiotensin receptor blockers; BB: beta-blockers; CCB: calcium channel blockers.

Variables	Normal	Osteopenia	Osteoporosis	P-value
Age of HTN diagnosis				
40–60 years	86 (46.7%)	66 (35.9%)	32 (17.4%)	0.56
Less than 40 years	17 (40.4%)	20 (47.6%)	5 (12%)	
More than 60 years	12 (41.4%)	10 (34.5%)	7 (24.1%)	
Antihypertensive drug				
ACE-I	13 (34.2%)	15 (39.5%)	10 (26.3%)	0.106
ARBs	9 (28.1%)	16 (50%)	7 (21.9%)	
BB	15 (75%)	4 (20%)	1 (5%)	
CCB	18 (41.9%)	15 (34.9%)	10 (23.3%)	
Thiazide	4 (50%)	3 (37.5%)	1 (12.5%)	
Antihypertensive drug				
Only one	59 (41.8%)	53 (37.6%)	29 (20.6%)	0.13
Two drugs combined	48 (53.9%)	30 (33.7%)	11 (12.4%)	

A regression analysis was conducted to investigate the relationship between demographic characteristics and HTN in OP patients. The analysis revealed that patients with increased age and parathyroid gland disorder had an adjusted OR (95% CI) of 1.17- and 15.7-fold higher risk (CI: 0.9-1.2, p-value = 0.048, and CI: 0.7-32, p-value = 0.04, respectively) of developing OP. Conversely, HTN patients with increased BMI had an adjusted OR (95% CI) of 0.9 (CI: 0.91 to 1.03, p-value = 0.03), corresponding to a 10% reduced risk, and literate patients had an adjusted OR of 0.1 (CI: 0.01-1.4, p-value = 0.046), corresponding to a 90% reduced risk of developing OP (Table 6).

**Table 6:** Risk factors of OP within demographic and clinical parameters in HTN patients Data were presented as odds ratio and 95% confidence interval. Multivariable logistic regression and odds ratio were calculated, adjusted for age, gender, BMI, marital status, smoking status, level of education, and comorbidities (diabetes, parathyroid gland disorders). p<0.05 significant variables are present. OR: odds ratio, CI: confidence interval, ref: reference, OP: osteoporosis, HTN: hypertension, BMI: body mass index.

Variables	Crude OR (95% CI)	p-value	Adjusted OR (95% CI)	p-value
Gender				
Female (ref. male)	2.24 (1.02–4.9)	0.04	3.1 (1.8–5.3)	0.06
Age in years (mean ± SD)	1.03 (0.9–1.07)	0.1	1.17 (0.9–1.2)	0.048*
BMI (mean ± SD)	0.98 (0.96–1)	0.049	0.9 (0.91–1.03)	0.03*
Menarche age (ref. more than 14 years)				
Less than 10 years	2.9 (0.59–14.4)	0.18	7.1 (0.08–12.1)	0.3
10 to 11 years	1.08 (0.4–2.5)	0.85	2.09 (0.04–2.4)	0.7
12 to 13 years	0.65 (0.14–2.9)	0.58	3.9 (0.07–6.2)	0.4
14 years	1.75 (0.46–6.6)	0.41	10.7 (0.9–102)	0.09
Menopause age (ref. 55 to 60)				
35 to 40 years	1.35 (0.3–5.7)	0.68	0.1 (0.007–2.1)	0.14
41 to 50 years	1.73 (0.40–7.3)	0.45	0.1 (0.01–1.7)	0.11
51 to 55 years	1.16 (0.1–6.5)	0.86	1.1 (0.04–1.9)	0.9
Marital status (ref. married)				
Single	0.56 (0.6–5.3)	0.6	0.8 (0.09–7.6)	0.7
Divorced	0.5 (0.23–1.07)	0.07	0.41 (0.19–1.3)	0.06
Widow	0.1 (0.03–0.15)	0.9	0.03 (0.006–0.1)	0.8
Smoking status				
Yes (ref. no)	1.25 (0.39–3.9)	0.69	1.48 (0.4–4.7)	0.5
Level of education (ref. university)				
Primary	0.93 (0.94 to −9.2)	0.95	0.04 (0.002–0.09)	0.6
Middle	0.55 (0.03–9.8)	0.68	0.3 (0.01–3.2)	0.9
High	0.5 (0.02–8.8)	0.63	1.7 (0.6–4.4)	0.4
Illiterate	3.07 (0.37 to −25.2)	0.29	1.5 (0.9–4.2)	0.3
Literate	2.1 (0.2–0.23)	0.5	0.1 (0.01–1.4)	0.046*
Comorbidities (ref. nothing)				
Diabetes	0.46 (0.30–6.5)	0.04	1.5 (0.5–4.4)	0.3
Parathyroid gland disorders	13.7 (1.1–22.8)	0.03	15.7 (0.7–32)	0.04*
Diabetes and parathyroid gland disorders	3.03 (1.23–7.32)	0.99	1.6 (0.5–4.4)	0.6

A regression analysis was performed between the clinical characteristics and HTN in OP patients. The analysis showed that HTN patients who were taking BB as their antihypertensive drug had a 0.23 adjusted OR (CI: 0.01-1.3, p-value:0.02), indicating a 77% reduced risk of developing OP. None of the other variables analyzed were statistically significant (Table 7).

**Table 7:** Risk factors of OP within clinical parameters in HTN patients Data were presented as odds ratio and 95% confidence interval. Multivariable logistic regression and odds ratio were calculated, adjusted for age of HTN diagnosis, antihypertensive drug use (ACE-I as reference), and drug combination (one drug as reference). p<0.05 significant variables are present. OR: odds ratio, CI: confidence interval, ref: reference, OP: osteoporosis, HTN: hypertension, ACE-I: angiotensin-converting enzyme inhibitors, ARBs: angiotensin receptor blockers, BB: beta-blockers, CCB: calcium channel blockers.

Variables	Crude OR (95% CI)	p-value	Adjusted OR (95% CI)	p-value
Age of HTN diagnosis (ref. 40–60 years)				
Less than 40 years	1.66 (0.84–3.27)	0.14	0.7 (0.2–2.6)	0.6
More than 60 years	1.29 (0.58–2.8)	0.5	0.8 (0.1–4.09)	0.8
Antihypertensive drugs (ref. ACE-I)				
ARBs	1.7 (0.66–4.3)	0.26	0.7 (0.2–2.3)	0.6
BB	0.15 (0.03–0.17)	0.009	0.23 (0.01–1.3)	0.02*
CCB	0.85 (0.34 to −2.7)	0.73	0.4 (0.07–2.1)	0.2
Thiazide	2.8 (0.69–11.2)	0.14	0.4 (0.04–3.7)	0.9
Drug combination				
Two drugs combined (ref. Only one)	0.83 (0.47–1.46)	0.53	2.1 (1.7–4.5)	0.85

## Discussion

OP and HTN are multifactorial diseases, and their association with each other contributes to considerable morbidity in the elderly population [12]. The relationship between HTN and OP is not clear yet. For that purpose, the risk factors for OP in HTN patients are discussed in the present study. HTN patients included in our study were regularly taking antihypertensive drugs to control blood pressure.

It has been observed that the rates of OP and HTN increase with life expectancy. However, some studies have reported no significant link between HTN and OP, indicating that HTN is not a risk factor for OP [6]. In contrast, other studies have demonstrated a connection between these two diseases, showing a high prevalence of OP among HTN participants [13,14]. Our study, conducted on individuals with HTN, detected notably the presence of osteopenia (38%) among HTN patients, with 16.9% being osteoporotic. These findings are consistent with a local Saudi study by Al-Hariri and Aldhafery, which reported notably the presence of OP among HTN patients, with osteopenia at 41.1% and OP at 27.8% [3]. Furthermore, based on these findings, our study agrees with the studies by Wu et al. and Hao Chai et al., which reported a high prevalence of OP among HTN patients [13,14]. In contrast, our results differ from those of Hijazi et al., who did not find an association between OP and HTN [6]. In our study, the ratio of OP was seen to be higher in elderly HTN patients, which confirms that OP is highly associated with increased age (mean age ± SD = 68.04 ± 7.60) [15]. We included both males and females of elderly age; among females, approximately all the females were postmenopausal. Our study determined that increased BMI reduced the risk of the development of OP in HTN patients by 10%; this is in agreement with the study, which showed a negative correlation between BMI and OP [16]. It was also observed that HTN patients with normal BMD were predominantly literate and had higher levels of education. In contrast, a significant increase in illiteracy was noted among HTN patients diagnosed with OP. We conducted multivariable logistic regression to investigate the further factors causing the increased risk. It has also been detected that in our study, HTN patients who are comorbid with DM are not associated with developing OP; this finding agrees with a previous study [17]. This study reported a prevalence of OP among diabetics of 6.4%. They found just 16 of 250 DM participants with severe OP [17]. On the other hand, our study also identified that HTN patients with parathyroid gland disorders increased the risk of developing OP by 15.1-fold. This finding aligns with existing literature suggesting a key role for parathyroid dysfunction in mediating the link between HTN and OP, as evidenced by studies [2,18].

Furthermore, we investigated the effect of antihypertensive drugs on the development of OP. HTN patients were taking only one medication or a combination of two medications. Regression analysis of our study showed that BB had an adjusted OR of 0.23, corresponding to a 77% reduction in the risk of developing OP, while no other antihypertensive drug induced or reduced the risk of OP in HTN patients. This finding agrees with the study by Hijazi et al. [6], which also reported the association of thiazide with an increased risk of OP [7]. However, our study did not find any significant association between these two.

Study limitations

This study has several limitations that should be considered. First, as a cross-sectional study, we are unable to establish a cause-and-effect relationship between hypertension and osteoporosis.

Second, we attempted to include additional risk factors such as vitamin D levels, calcium levels, and lipid profiles. However, we found that over 50% of the participants did not have available data for vitamin D and calcium levels. Similarly, lipid profile data were missing for approximately 40% of the participants. Even when available, these measurements were recorded using different units, complicating comparisons and analyses.

Lastly, 255 participants were included in the study. Among them, 141 (55.3%) were taking only one antihypertensive medication, while 89 (34.9%) were on a combination of antihypertensive drugs. Unfortunately, complete drug history data were missing for 25 participants (9.8%) due to missing medical records. Despite our best efforts to locate their most recent prescriptions, we were unable to gather enough information for this group.

These limitations highlight the challenges inherent in conducting research with elderly populations and underscore the need for careful consideration of data completeness and consistency in future studies.

Special recommendations

First: To Healthcare Practitioners

Based on the identified risk factors (e.g., age, gender, educational level, and comorbidities), it is recommended that healthcare practitioners screen HTN patients for OP risk factors and consider BMD testing for HTN patients who are illiterate or have specific comorbidities (e.g., parathyroid disorders).

Second: To Policymakers

We propose community health strategies to raise awareness and educate the community about OP prevention among HTN individuals: develop educational programs targeting HTN patients and healthcare providers about the link between HTN and OP. Collaborate with public health authorities and organizations to integrate OP screening into routine HTN patient care protocols and advocate for policies that support preventive healthcare measures for bone health in HTN patients, including access to screening and treatment options.

## Conclusions

The study findings indicate that OP is notably present among patients with HTN, particularly in older individuals. Additionally, individuals with parathyroid gland disorders exhibited a markedly increased risk, with a 15.1-fold higher likelihood of developing OP. In contrast, increased BMI and the use of BB as an antihypertensive medication are associated with a reduced risk of OP in this population. Any level of education above illiteracy is associated with a lower prevalence of OP, suggesting that education may have a protective effect against OP in HTN patients. No significant association was found between DM and the development of OP in HTN patients. However, we recommend more studies to know more about the prevalence and associated risk factors of OP in HTN patients in Saudi Arabia. Our proposed research in the future includes determining the prevalence of OP among HTN patients in different age groups and genders, assessing the impact of educational levels and socioeconomic factors on OP prevalence, and investigating the association between specific comorbidities (e.g., DM, parathyroid gland disorders). For healthcare providers, we recommend screening HTN patients, especially those of older age, for OP risk factors. BMD testing might be considered for an illiterate population or have specific comorbidities (e.g., parathyroid disorders). To address this public health concern, policymakers should consider educational programs and integrated screening protocols for HTN patients. Additionally, advocating for policies that improve access to preventive bone health measures is crucial.

## Appendices

Appendix 1

Prevalence and Associated Factors of Osteoporosis Among People With Hypertension in Al-Ahsa, Saudi Arabia:

As part of our research objectives to explore the prevalence and associated factors of osteoporosis among hypertension patients in Alahsa, we kindly request your participation in this questionnaire. Osteoporosis is a condition characterized by decreased bone density and an increased risk of fractures, especially among individuals with hypertension. Understanding the prevalence and factors associated with osteoporosis can greatly inform healthcare strategies and improve patient outcomes. Your valuable participation will contribute to advancing our understanding of this important health issue. Thank you for your participation.

Participant consent

·       Yes, I agree.

·       No.

General health

Age of menarche:

·       Not applicable.

·       Less than 10 years.

·       10 to 11 years.

·       12 to 13 years.

·       14 years.

·       More than 14 years.

Age of menopause:

·       Not applicable.

·       35 to 40 years.

·       41 to 50 years.

·       51 to 55 years.

·       55 to 60 years.

·       Not yet.

Which age were you diagnosed with hypertension?

·       Less than 40 years.

·       40 to 60 years.

·       More than 60 years

Comorbidities

·       Diabetes.

·       Parathyroid gland disorders.

·       Renal diseases.

·       Cancer.

·       Nothing.

Lifestyle factors

Smoking status:

·       Yes.

·       No.

Socioeconomic level

What is your occupation?

·       Governmental employee.

·       Private employee.

·       Business owner.

·       Retired.

·       Housewife.

·       I prefer not to answer.

What is your education level?

·       Illiterate.

·       Literate.

·       Primary school.

·       Middle school.

·       High school.

·       University.

·       I prefer not to answer.

Marital status:

·       Single.

·       Married.

·       Divorced.

·       Widowed.

End of the interview.

Thank you for your participation.
